# Cocaine Addiction Treatments to improve Control and reduce Harm (CATCH): New Pharmacological Treatment Options for Crack-Cocaine Dependence in the Netherlands

**DOI:** 10.1186/1471-244X-11-135

**Published:** 2011-08-19

**Authors:** Mascha Nuijten, Peter Blanken, Wim van den Brink, Vincent Hendriks

**Affiliations:** 1 Parnassia Addiction Research Centre (PARC, Brijder Addiction Treatment), PO Box 53002, 2505 AA The Hague, the Netherlands; 2 Amsterdam Institute for Addiction Research, Department of Psychiatry, Academic Medical Centre, University of Amsterdam, PO Box 22660, 1100 DD Amsterdam, the Netherlands

## Abstract

**Background:**

Cocaine, particularly in its base form ('crack'), has become one of the drugs of most concern in the Netherlands, being associated with a wide range of medical, psychiatric and social problems for the individual, and with significant public order consequences for society. Available treatment options for cocaine dependent users are limited, and a substantial part of the cocaine dependent population is not reached by the addiction treatment system. Psychosocial interventions for cocaine dependence generally show modest results, and there are no registered pharmacological treatments to date, despite the wide range of medications tested for this type of dependence.

The present study (Cocaine Addiction Treatments to improve Control and reduce Harm; CATCH) investigates the possibilities and problems associated with new pharmacological treatments for crack dependent patients.

**Methods/Design:**

The CATCH-study consists of three separate randomised controlled, open-label, parallel-group feasibility trials, conducted at three separate addiction treatment institutes in the Netherlands. Patients are either new referrals or patients already in treatment. A total of 216 eligible outpatients are randomised using pre-randomisation double-consent design and receive either 12 weeks treatment with oral topiramate (n = 36; Brijder Addiction Treatment, The Hague), oral modafinil (n = 36; Arkin, Amsterdam), or oral dexamphetamine sustained-release (n = 36; Bouman GGZ, Rotterdam) as an add-on to cognitive behavioural therapy (CBT), or receive a 12-week CBT only (controls: n = 3 × 36).

Primary outcome in these feasibility trials is retention in the underlying psychosocial treatment (CBT). Secondary outcomes are acceptance and compliance with the study medication, safety, changes in cocaine (and other drug) use, physical and mental health, social functioning, and patient satisfaction.

**Discussion:**

To date, the CATCH-study is the first study in the Netherlands that explores new treatment options for crack-cocaine dependence focusing on both abstinence and harm minimisation. It is expected that the study will contribute to the development of new treatments for one of the most problematic substance use disorders.

**Trial Registration:**

The Netherlands National Trial Register NTR2576

The European Union Drug Regulating Authorities Clinical Trials EudraCT2009-010584-16

## Background

Cocaine, particularly in its base form ('crack'), has become one of the drugs of most concern in many countries, being associated with a wide range of medical, psychiatric and social problems for the individual, and with significant public order consequences for society [1-3]. In the Netherlands (16.7 million inhabitants), annual cocaine-related addiction treatment demand increased from 8,490 patients in 1994 to 17,270 in 2008, and approximately half of this treatment demand in 2008 concerned users of crack [4]. Although the number of treatment seeking cocaine users slightly decreased in more recent years, the number of cocaine dependent patients that repeatedly returned to addiction treatment increased [5]. Despite its status as one of the most problematic addictions, reliable prevalence estimates of cocaine dependence in the Netherlands are lacking. In 2005, 32,000 current cocaine users were identified in the Netherlands [4], but this is likely to be a serious underestimation given the fact that the data were obtained in a population survey. Moreover, the survey did not allow a separate estimate of the prevalence of crack cocaine use.

Psychosocial treatments for cocaine dependence, which include cognitive behavioural therapy, counselling and relapse prevention, have generally produced modest results [6,7], and both study data and practice-based experiences indicate that poor compliance is a major complicating factor in these treatments. One of the more promising psychosocial treatments for cocaine dependence to date is contingency management, which has shown positive results in terms of improved treatment retention and reduction of substance use in a series of studies [7-11], and is therefore currently being investigated in the Netherlands in the context of a controlled study in heroin addicts with concurrent cocaine use. However, dissemination of contingency management has been problematic because of low acceptance and limited experience of therapists with this intervention [12] and because contingency management is politically controversial because communities are often not willing to just pay for a change in health behaviour [13].

The modest results of psychosocial treatments and the increasing knowledge about the neurobiology of cocaine dependence have led to an increasing number of studies searching for effective pharmacological agents that influence the neurochemistry of cocaine, including antipsychotics, anticonvulsants, antidepressants, psychostimulants and (other) dopamine agonists [14-22]. Despite the considerable efforts in this field, however, there are no proven effective pharmacotherapies for cocaine dependence to date, and the testing of new medications for cocaine dependence should continue to be high on the research agenda. Basically, the research efforts are focused on two pharmacological strategies [23]: one directed at abstinence from - or at least substantial reduction of - cocaine use and the other directed at minimizing cocaine-related harm by replacing short-acting, illicit cocaine by a long acting, legal stimulant that can be taken orally [24-26].

Concerning the first strategy, from the wide range of medications tested, topiramate and modafinil are examples of new medications that are currently only registered for indications other than cocaine dependence, but have shown promise in several studies in cocaine dependent populations in terms of abstinence or stimulant use reduction [16,27,28].

Topiramate was originally marketed as an anticonvulsant. Through its effects on the GABA- and the glutamate-system, it attenuates dopamine neurotransmission. In the alcohol field, various randomised controlled trials have shown that topiramate was more effective than placebo in treating alcohol dependence [29-31], and was at least as effective as naltrexone [29,30]. Concerning cocaine, topiramate was more effective in promoting abstinence and sustained abstinence in (crack-) cocaine users in a double-blind placebo-controlled pilot trial of Kampman and colleagues [32], and cocaine craving significantly decreased after the administration of topiramate in an open label trial [33]. A second promising treatment option is the use of the alpha-adrenergic/glutamate agonist modafinil, which is generally prescribed for the treatment of narcolepsy, obstructive sleep apnoea/hypopnoea and shift work sleep disorder. In addition, modafinil showed effectiveness in terms of duration of abstinence in the treatment of cocaine dependence in two randomised controlled trials [34,35], and in the reduction of craving in cocaine dependent patients without comorbid alcohol dependence [36]. More recently, modafinil was investigated in methamphetamine dependence with improved treatment retention and decreased methamphetamine use as a result [37-40].

With respect to the second strategy, harm reduction or drug use reduction oriented treatment, a growing number of pre-clinical and human studies suggest that the monoamine releaser dexamphetamine, used for the treatment of attention deficit hyperactivity disorder (ADHD) and narcolepsy, is an important candidate for replacement therapy [18,28]. The basic rationale for substitution treatment for cocaine dependence is similar to that for other addictions (nicotine replacement therapy in nicotine dependence, methadone and buprenorphine in opioid dependence): it aims to replace uncontrolled and harmful drug use with regulated and safer use, in terms of dose, route of administration and adverse effects, and to facilitate engagement with health care services by attracting and retaining addicted individuals in treatment [41,42]. In addition, the regular supervised prescription regimen may by itself help patients to structure their daily life. In cocaine dependent patients several controlled studies have shown significant improvements associated with the administration of sustained-release (SR) dexamphetamine, without serious adverse events (including no serious cardiovascular complications). Shearer and colleagues [43] reported positive results of dexamphetamine SR in a placebo-controlled study of cocaine dependent injectors in terms of reduced cocaine use, craving, severity of dependence and delinquent behaviour, and dexamphetamine SR was found to attenuate cocaine use and improve treatment retention in combined cocaine and heroin dependent patients in controlled studies of Greenwald et al. [44] and Grabowski et al. [45,46].

In sum, cocaine dependency is characterized by its chronic and relapsing nature and by high treatment dropout rates. A wide range of pharmacological agents has been tested for efficacy in cocaine dependence, but generally with disappointing or, at best, equivocal results. From the investigated candidate medications, topiramate, modafinil and dexamphetamine SR have shown the most promising results. The vast majority of these studies were conducted in the US, however, and therefore these study findings need to be confirmed in research outside the US.

The overall objective of the current study is to investigate topiramate, modafinil, and dexamphetamine SR for their acceptability and effectiveness in the treatment of cocaine dependent patients in The Netherlands. Dependent on the results, this study will also yield candidate medications for further investigation in a large-scale confirmatory trial. More specifically, we aim to evaluate in three separate randomised controlled, open-label, parallel-group feasibility trials in crack-cocaine dependent patients the response to each of these three medications, as an add-on to psychosocial treatment with cognitive behavioural therapy (CBT), compared to CBT alone, in terms of acceptance, treatment retention and compliance, efficacy, safety, and patient satisfaction. As in any medication study, our primary focus is on the balance between (potential) benefit and harm associated with the medications, taking into consideration the personal and societal damage linked to continued illicit use of cocaine, in a situation without effective pharmacological treatment options.

Because of the aim of the study - investigating treatment effectiveness with both abstinence and harm minimisation as treatment strategies for cocaine dependent patients - the study is named CATCH: Cocaine Addiction Treatments to improve Control and reduce Harm.

## Methods/Design

### Research design

In each of the three sub-studies, patients are randomly assigned to an experimental or control condition. Patients in both conditions receive basic psychosocial treatment in the form of outpatient cognitive-behavioural therapy (CBT), and those in the experimental group receive pharmacotherapy with one of the proposed medications as an add-on to CBT.

In the largest series of studies of pharmacological treatment options for cocaine dependence conducted to date, the Cocaine Rapid Efficacy Screening Trials (CREST), a randomised, controlled, parallel group, double-blind design with an unmatched placebo was used in all studies [47]. However, dropout rates in the control groups were observed earlier during the active treatment period, probably caused by inefficacy, and may have biased outcomes (see [15,48]).

To avoid premature dropout by disappointment about the absence of pharmacological effects in the control groups, we decided not to use the conventional double-blind placebo-controlled design. Instead, we opted for a design in which patients in the control conditions are unaware of the comparative experimental condition in which patients are prescribed active medication. Central feature of this so-called pre-randomisation (or 'Zelen') design [49] is that randomisation takes place prior to seeking (final) informed consent. The variant we use is the so-called double-consent design. Compared to the conventional randomised design, the pre-randomisation double-consent design offers the advantage of providing a more naturalistic control condition, without information or selection bias due to patients being aware that they are control subjects, but with the strengths of a randomised design. The double-consent design is considered particularly useful if the experimental intervention is expected to be attractive to the participants, which is likely to result in considerable disappointment, non-compliance and loss to follow-up among control subjects in a conventional randomised design.

**Figure 1:**
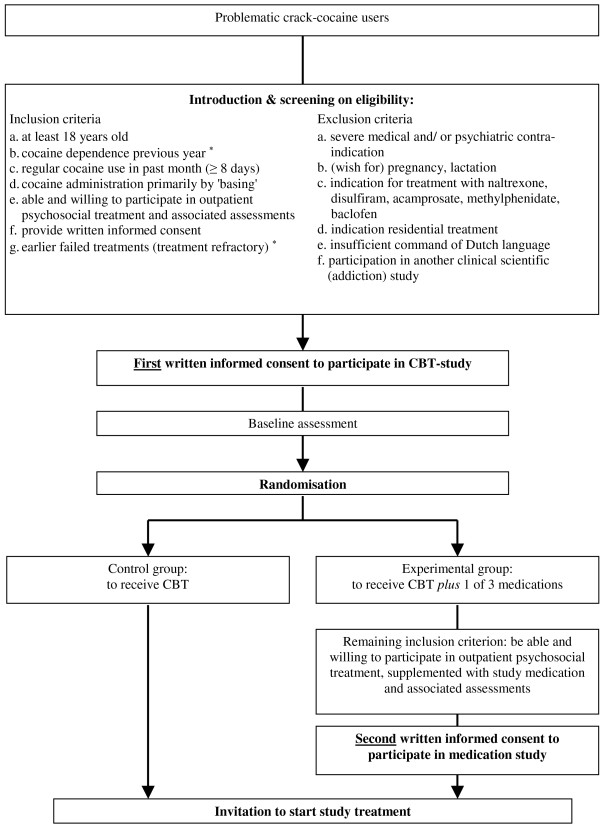
**Flowchart inclusion patients with respect to double-consent selection procedure**. * Patients participating in the dexamphetamine study must have a minimal duration of cocaine dependency of five years and a history of earlier failed treatments aimed at reducing or abstaining from cocaine use.

All study participants are asked to provide informed consent to participate in the psychosocial treatment (CBT). After obtaining the (first) informed consent, these patients are randomised to the experimental or control condition, and only those in the experimental condition are asked to provide a second informed consent to participate in the additional pharmacological treatment. Hence, patients are only informed about the assigned treatment, but not about the comparison treatment. In Figure 1 the stepwise procedure according to the pre-randomisation double-consent design is displayed.

This study was approved by the Medical Ethics Committee of the Academic Medical Centre in Amsterdam, the Netherlands (protocol number MEC 09/197).

### Participants - setting

Since the use of crack-cocaine is most prevalent in the urban western areas of the Netherlands, the three sub-studies are executed at treatment sites in the three largest Western cities. In The Hague, topiramate is investigated at Brijder Addiction Treatment; in Amsterdam, modafinil is investigated at Arkin; and in Rotterdam, dexamphetamine SR is investigated at Bouman GGZ.

In all three sub-studies, eligible patients must (a) be at least 18 years old, (b) be cocaine dependent (DSM-IV) during at least the previous year, (c) use cocaine on a regular basis (i.e., ≥ 8 days) in the previous month, (d) administer their illicit cocaine primarily by means of basing ('crack'), (e) be able and willing to participate in outpatient psychosocial treatment and associated assessments (control condition), or be able and willing to participate in outpatient psychosocial treatment supplemented with the study medication and associated assessments (experimental condition), and (f) have provided written informed consent with regard to their assigned study treatment.

Patients are excluded in case of (a) severe medical (e.g., severe renal insufficiency; cardiovascular problems) or severe psychiatric problems (e.g. acute psychosis, current major depression, suicidality) that constitute a contra-indication for participation, (b) pregnancy or continued lactation, (c) indication for treatment with naltrexone, disulfiram, acamprosate, methylphenidate or baclofen, (d) indication for residential treatment (clinical judgment), (e) insufficient command of the Dutch language, and (f) current participation in another addiction treatment trial.

Given the nature of the medication and the social debate on the use of dexamphetamine for the treatment of cocaine dependence, in the sub-study with dexamphetamine SR, the required duration of cocaine dependency is at least five years instead of one year. Furthermore, these patients have to meet one additional inclusion criterion: (g) a history of at least two failed treatments directed at reduction of or total abstinence from cocaine use ('treatment-refractory').

### Recruitment & Screening

Study participants are either new referrals to the addiction treatment service with cocaine dependence as their main problem or patients with cocaine dependence already in treatment for another type of dependence, e.g. methadone treatment for opioid dependence.

There are two eligibility screening sessions. In the first session, the CATCH-study is explained and initial eligibility criteria are examined. Study information and informed consent forms are given to read before the second screening session generally one week later. In addition, baseline measurements are conducted.

In the second session, final eligibility is determined by a physician, based on the health and medical status. Eligible patients that agree to participate in the outpatient psychosocial treatment (CBT) and study assessments are asked to provide written informed consent. After this (first) informed consent is obtained, patients are randomised to one of the treatment conditions, i.e. with or without pharmacotherapy. Patients in the experimental group are then notified about the possibility to receive pharmacological treatment as an add-on to CBT, and are informed about the study medication and associated study procedures. Only from these patients, a second informed consent is obtained in a follow-up meeting with the physician, usually one week later.

### Psychosocial treatment

CBT in the experimental and control condition incorporates a combination of outpatient cognitive behavioural counselling, relapse prevention and motivational interviewing, which is considered a standard substance abuse treatment in the Netherlands ("Leefstijltraining") [50]. CBT is delivered in 12 weekly individual sessions of 45-minutes by an experienced psychologist who received formal training in CBT for the purpose of the trial. Dependent on the treatment goal of the individual patient, treatment is directed at stabilisation and harm reduction, reduction of cocaine use, or total abstinence.

### Pharmacological treatments

Each of the three proposed medications is prescribed for a period of 12 weeks as an add-on to CBT in the experimental group. Topiramate is titrated within three weeks to a maximum oral dose of 200 mg/day, depending on the profile of adverse events observed. During the first treatment week, topiramate is prescribed on a daily basis at the treatment centre. In the remaining trial period, topiramate is dispensed to the patient once a week, i.e. with take-home doses for a maximum of seven days.

Modafinil is prescribed in a fixed oral dose of 200 mg/day during the first treatment week and in a maximum dose of 400 mg/day during the remaining treatment weeks. The prescription regimen of modafinil is similar to that of topiramate (see above).

Dexamphetamine sulphate is prescribed in sustained-release (SR) form, in a daily, oral fixed, dose of 60 mg. To allow more intense safety monitoring and to prevent diversion, dexamphetamine is prescribed on a daily basis at the treatment centre during the first four weeks of the trial period. In the remaining trial period, dexamphetamine is dispensed twice a week (take home doses for a maximum of three days). In addition, during the first four weeks daily assessments of heart rate and blood pressure are conducted, whereas in the remaining treatment period assessments of heart rate and blood pressure take place two times per week.

### Assessments and instruments

Study assessments take place at baseline and at four, eight (experimental group only) and 12 weeks (endpoint) following baseline. Table 1 gives an overview of the instruments to be applied at baseline and follow-up assessment points.

**Table 1:** Overview of assessments and instruments

Assessments (researchers)	Baseline	Week 4^•^	Week 8^•^	Week 12
Checklist inclusion criteria (control and experimental)	•			
CIDI - Substance Abuse Module (cocaine& heroine& alcohol)	•			
MINI - Suicidal risk	•			
Addiction Severity Index + supplement^1^	•			
Addiction Severity Index; short version substance use + supplement^1^		•	•	•
Time Line Follow-Back (cocaine)	•	•	•	•
Maudsley Addiction Profile (MAP) - HSS	•	•	•	•
Symptom Check List- 90 (SCL-90)	•	•	•	•
Obsessive Compulsive Drug Use Scale (OCDUS)	•	•	•	•
Client Satisfaction Questionnaire- 8 (CSQ-8) + supplement^2^				•
Evaluation (control and experimental)				•
				

**Medical assessment (physician)**	**Baseline**	**Week 4^•^**	**Week 8^•^**	**Week 12**

Checklist exclusion criteria (control and experimental)	•			
First informed consent (control and experimental)	•			
Second informed consent (only experimental)	•			
Medical assessment: ECG (only dexamphetamine study)	•			•
Medical assessment: blood analysis (week 12 only experimental)	•			•
Medical screening: heart rate, blood pressure^3 ^(only experimental)	•	• • • •	• • • •	• • • •
Pregnancy (only experimental; only women)	•	•	•	•
(Serious) adverse events (only experimental)	•	• • • •	• • • •	• • • •
Drug accountability (only dexamphetamine study)		• • • •	• • • •	• • • •
Urinalysis cocaine- metabolites (control and experimental; obtained by research assistant)	•			• • • •
Treatment participation (consumption of care; medication) (control and experimental)		• • • •	• • • •	• • • •

*Note*. ^• ^The assessments in week 4 and 8 are only for participants in the experimental groups (topiramate, modafinil, dexamphetamine SR), ^1 ^The ASI-supplement concerns illegal activities, income, and living arrangements, ^2 ^Additional questions about the treatment(s), ^3 ^In the dexamphetamine study, blood pressure and heart rate will be assessed every day during the first four weeks, • • • • Indicates weekly (instead of monthly) registration.

At baseline, the Composite International Diagnostic Interview Substance Abuse Module (CIDI-SAM) [51] is used to obtain DSM-IV [52] past year diagnoses of cocaine use disorder and other substance use disorders, the subsection 'suicidal risk' of the Mini-International Neuropsychiatric Interview (MINI) to determine possible suicidality [53-55], and the Addiction Severity Index (ASI) [56] to assess baseline demographics and clinical characteristics.

At baseline and all subsequent time points, the Time Line Follow-Back (TLFB) calendar method [57] is administered to collect detailed information about the patients' self-reported cocaine use during the period preceding each assessment. The short version of the Obsessive Compulsive Drug Use Scale (OCDUS) is used to measure cocaine craving [58-60], the Maudsley Addiction Profile-Health Symptoms Scale (MAP-HSS) [61] to assess physical status, and the Symptom Checklist-90 (SCL-90) [62] to identify psychological problems.

All assessments following baseline are supplemented with substance use items of the ASI and questions about current illegal activities, income, and living arrangements.

At the end of the study period (week 12), the Client Satisfaction Questionnaire (CSQ-8) [63], supplemented with questions about the received psychosocial and pharmacological (experimental groups only) treatment is added to assess patient satisfaction. Treatment adherence is defined as the number of attended CBT sessions.

All these assessments are administered by specially trained research assistants.

Medical assessments are conducted by a physician or laboratory personnel and include: blood pressure, heart rate, blood tests, ECG (dexamphetamine study only) and a pregnancy test (women only) for all participants at baseline. Participants of the experimental groups also receive a weekly medical screening (dexamphetamine study daily in the first four weeks), including blood pressure and heart rate, and women are tested for pregnancy monthly (see Table 1). At week 12, medical status of all participants is evaluated, including blood tests and ECG (dexamphetamine study only) for the experimental groups.

Urine samples are collected by research assistants at baseline, and at the four weeks preceding the endpoint assessment, and are analysed on the presence of benzoylecgonine (BE).

### Remuneration

Patients receive a remuneration for their participation: €30 for the baseline assessment, €15 for follow-up assessments after four and eight weeks (experimental groups only), and €30 for the endpoint assessment after 12 weeks. In addition, patients receive €15 for each urine sample provided during the four weeks preceding endpoint assessment. The maximum remuneration is €150 in the experimental group and €120 in the control group.

### Outcome measures and data analyses

The study data are analysed following an intent-to-treat (ITT) approach. The ITT-population consists of all patients who provide informed consent pertaining to the assigned treatment.

The primary outcome measure is treatment retention, defined as the number of attended CBT sessions following the start of treatment. In each sub-study, treatment groups are compared on duration of treatment until (premature or planned) termination, using a Cox proportional hazards regression model. Compliance with the study medication can only be investigated in the experimental treatment conditions.

Secondary effects of the interventions are evaluated in terms of treatment compliance, safety, cocaine use (self-report; urinalyses), cocaine craving, use of other substances, physical and mental health, social functioning (including criminality), and patient satisfaction. Pre- to post-treatment changes in days of cocaine use from baseline to week 12 are compared between the two study groups in each sub-study using a 2 (time) × 2 (group) repeated measures ANOVA. Similar analyses are conducted for changes in substance use other than cocaine, and for other continuous and Likert-scaled outcome measures.

Concerning the results of urinalyses, the outcome measure is the sum of BE negative urine samples during the four weeks preceding the endpoint assessment considering missing urine samples as positive. Negative BE urines are compared between the two study groups and evaluated for significant differences by means of an analysis of variances model with the baseline urine sample as covariate.

Following the analyses pertaining to each of the three sub-studies, the datasets from the sub-studies are pooled to further explore the prognostic value of various patient baseline characteristics with increased power.

### Safety

Adverse events (AEs) are defined as any undesirable medical experience occurring to a study subject during the study. All AEs reported spontaneously by the subject or observed by the treating physician or his staff are registered on a weekly basis by the treating physician, defining the relationship of each AE with the study medication (definitely not, possibly, probably, certainly, unknown), and its severity. If the AE is considered severe, it is registered as a serious adverse event (SAE). All AEs will be followed until they have abated, or until a stable situation has been reached. If the AE is related to the study medication, it is defined as an adverse reaction (AR) or - if severe - a serious adverse reaction (SAR). An AR can be unexpected when the nature or severity is not consistent with the information in the study medications' summary of product characteristics. SAEs and SARs will be annually reported to the medical ethical committee and the medicine evaluation board.

SAEs that are both unexpected and at least possibly related to the study medication are defined as suspected unexpected serious adverse events (SUSARs). SUSARs that arise during the study are reported within 15 days after the first knowledge of the SUSAR, or within 7 days in case of a fatal or life-threatening SUSAR to the medical ethical committee and the medicine evaluation board of the Netherlands.

### Power considerations

In the primary analysis, treatment effect is investigated by means of a Cox proportional hazards regression model. With a difference in 12-week survival between the medication and control groups of 25% (i.e. 45% vs. 20%; hazard rate of 0.066 in the experimental condition vs. 0.135 in the control condition), power of 0.80, and two-sided alpha = 0.10, 36 patients are required in each study group (2 × n = 36 in each medication sub-study; total n = 3 × 2 × 36 = 216 patients). Given the feasibility character of the study, as well as to enable testing of various medications in one study, the sample sizes per trial are necessarily limited. As in the earlier mentioned CREST project, a lenient alpha of 0.10 was chosen to minimize loss of statistical power due to small sample sizes, instead of the usual 5% false-positive rate, which would be more appropriate for future confirmatory trials. Moreover, some earlier studies of modafinil and dexamphetamine SR in cocaine addicts found significant results with comparable sample sizes [34,43,45,46].

## Discussion

Given the considerable number of problematic crack-cocaine users and the lack of effective psychosocial and pharmacological treatments, the CATCH-study is the first study in the Netherlands that explores new pharmacological treatment options for this type of addiction that are not only focused on abstinence (CBT with or without topiramate or modafinil), but also on harm minimization using an agonist approach (CBT with or without dexamphetamine SR). It is expected that the study will contribute to opening up new lines of research and - dependent upon the results - new lines of treatment for one of the most problematic substance use disorders: crack-cocaine dependence.

A limitation of this study is that control patients will not receive placebo (in addition to their CBT) and that experimenters' bias towards the medication group can not be eliminated due to the open-label design. However, given the high dropout rates in controls such as in the CREST-studies, these disadvantages are defendable in the context of the proposed feasibility trials.

## Competing interests

The authors declare that they have no competing interests.

## Authors' contributions

VH and PB created the design of the study and composed the request for funding together with WvdB. MN drafted this paper. All authors revised this manuscript and approved the final version.

## Pre-publication history

The pre-publication history for this paper can be accessed here:

http://www.biomedcentral.com/1471-244X/11/135/prepub
